# Prospective evaluation of an internet-linked handheld computer critical care knowledge access system

**DOI:** 10.1186/cc2967

**Published:** 2004-10-14

**Authors:** Stephen E Lapinsky, Randy Wax, Randy Showalter, J Carlos Martinez-Motta, David Hallett, Sangeeta Mehta, Lisa Burry, Thomas E Stewart

**Affiliations:** 1 Director, Technology Application Unit and Site Director, Intensive Care Unit, Mount Sinai Hospital & Interdepartmental Division of Critical Care, University of Toronto, Toronto, Ontario, Canada; 2 Director, Human Simulation, Technology Application Unit and Intensive Care Unit, Mount Sinai Hospital & Interdepartmental Division of Critical Care, University of Toronto, Toronto, Ontario, Canada; 3 Research Coordinator, Technology Application Unit, Intensive Care Unit, Mount Sinai Hospital, Toronto, Ontario, Canada; 4 Biostatistician, Intensive Care Unit, Mount Sinai Hospital, Toronto, Ontario, Canada; 5 Research Director, Intensive Care Unit, Mount Sinai Hospital & Interdepartmental Division of Critical Care, University of Toronto, Toronto, Ontario, Canada; 6 ICU Pharmacist, Intensive Care Unit, Mount Sinai Hospital, Toronto, Ontario, Canada; 7 Director of Critical Care, Mount Sinai Hospital and University Health Network & Interdepartmental Division of Critical Care, University of Toronto, Toronto, Ontario, Canada

**Keywords:** clinical, computer, critical care, decision support systems, handheld, internet, point-of-care systems, practice guidelines, simulation

## Abstract

**Introduction:**

Critical care physicians may benefit from immediate access to medical reference material. We evaluated the feasibility and potential benefits of a handheld computer based knowledge access system linking a central academic intensive care unit (ICU) to multiple community-based ICUs.

**Methods:**

Four community hospital ICUs with 17 physicians participated in this prospective interventional study. Following training in the use of an internet-linked, updateable handheld computer knowledge access system, the physicians used the handheld devices in their clinical environment for a 12-month intervention period. Feasibility of the system was evaluated by tracking use of the handheld computer and by conducting surveys and focus group discussions. Before and after the intervention period, participants underwent simulated patient care scenarios designed to evaluate the information sources they accessed, as well as the speed and quality of their decision making. Participants generated admission orders during each scenario, which were scored by blinded evaluators.

**Results:**

Ten physicians (59%) used the system regularly, predominantly for nonmedical applications (median 32.8/month, interquartile range [IQR] 28.3–126.8), with medical software accessed less often (median 9/month, IQR 3.7–13.7). Eight out of 13 physicians (62%) who completed the final scenarios chose to use the handheld computer for information access. The median time to access information on the handheld handheld computer was 19 s (IQR 15–40 s). This group exhibited a significant improvement in admission order score as compared with those who used other resources (*P *= 0.018). Benefits and barriers to use of this technology were identified.

**Conclusion:**

An updateable handheld computer system is feasible as a means of point-of-care access to medical reference material and may improve clinical decision making. However, during the study, acceptance of the system was variable. Improved training and new technology may overcome some of the barriers we identified.

## Introduction

The rate of expansion of medical knowledge is increasing rapidly, and it is frequently difficult for clinicians to keep abreast of important new literature. For example, several recently published randomized controlled trials in critical care have demonstrated mortality benefits [1-5], but uptake of new knowledge into clinical practice is often delayed [6-8]. Improving access to this knowledge base at the point of care may lead to better clinical decision making, which could improve patient outcome, reduce costs and optimize bed utilization [9]. In critical care, rapid access to medical reference information may be particularly important in facilitating timely management decisions and avoiding errors [10].

Computing technology can allow point-of-care access to up-to-date medical reference material [11]. A study evaluating a mobile computerized cart to make evidence available to clinicians in an internal medicine setting [12] demonstrated that evidence-based medicine was more likely to be incorporated into patient care when the computerized system was used. Because of their portability, handheld devices may be more practical tools for disseminating knowledge to the point of care. Despite the popularity of handheld devices in medicine, few studies have evaluated the usefulness of this technology [13]. Before widespread dissemination of this type of technology can be encouraged, its impact must be thoroughly evaluated [14]. In the present study we evaluated whether it would be feasible and effective to provide updateable reference information from a central academic centre to handheld computers used by critical care specialists in community hospitals.

## Methods

### Study design, participants and setting

A total of 17 intensivists at four community hospital intensive care units (ICUs) in the Greater Toronto Area participated in the present prospective interventional study.

### Intervention

After training, each physician was equipped with a handheld computing device (Palm M505; Palm Inc., Milpitas, CA, USA) loaded with medical reference material pertinent to the critical care physician. This information included a customized critical care information handbook ('Critical Care'), which was previously developed for use by residents and physicians at our centre (Additional file 1). Commercially available medical reference software was also incorporated, namely PEPID ED (PEPID LLC, Skokie, IL, USA) and MedCalc .

**Figure 1:**
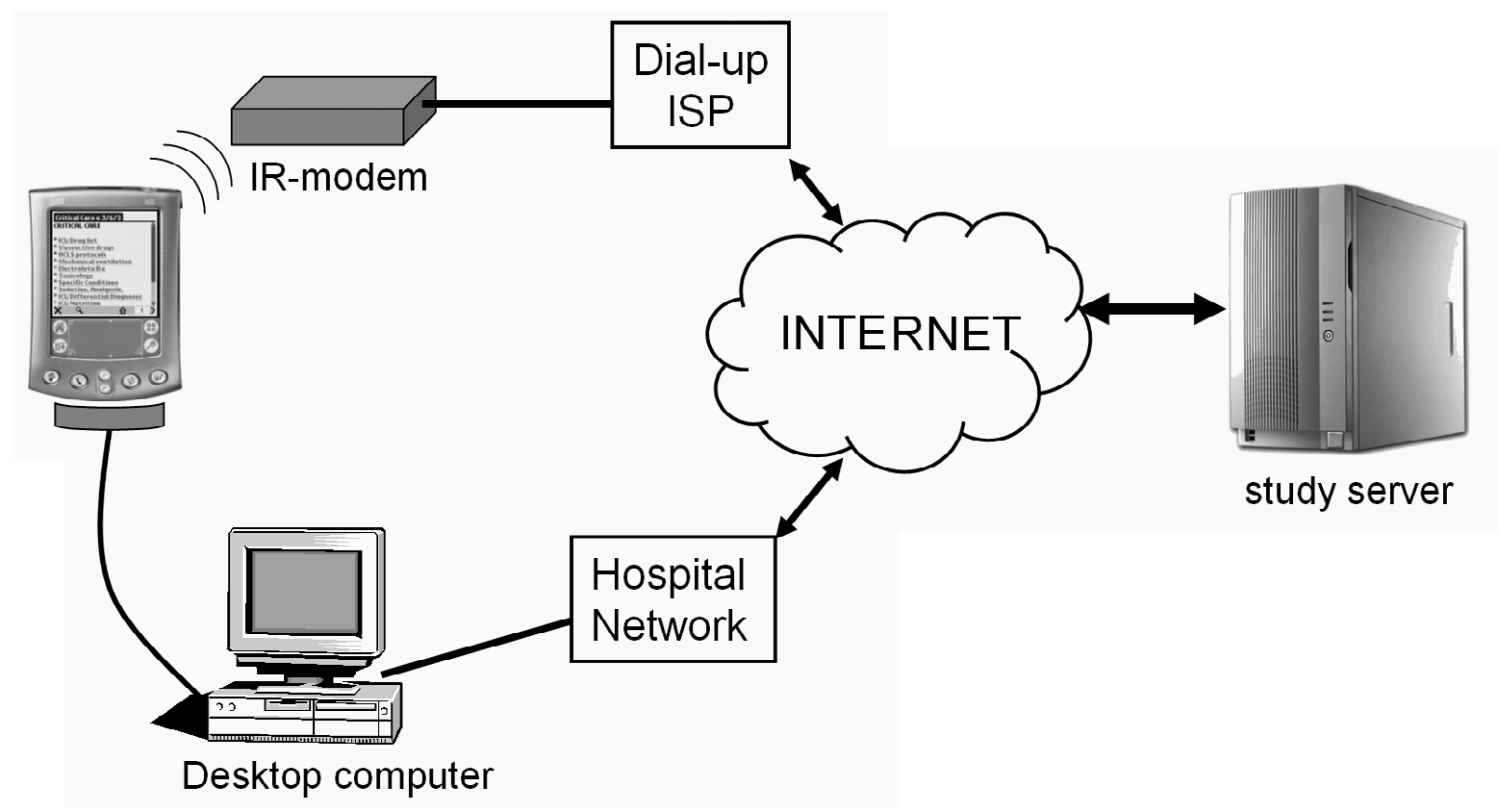
The internet-based data transfer system. Updated information is downloaded to the handheld device from a study server. Connection to the internet can take place via hardwire synchronization with a desktop computer or using infrared (IR) data transmission to a dial-up modem. ISP, internet service provider.

The handheld devices were able to receive literature updates on a regular basis, using customized software (IqSync; Infiniq Software, Mississauga, Ontario, Canada), which accessed an internet-based server using either a connection via desktop computer or infrared data transfer to a telephone modem (Fig. 1). New information was sent to the handheld devices and appeared in a file called 'What's New'. These updates, provided every 2–3 weeks, comprised brief reviews of relevant new literature including a short summary, a commentary and the article abstract.

All handheld devices were equipped with backup software that allowed the content to be rapidly restored in the event of a hardware failure (BackupBuddy VFS; Blue Nomad Software, Redwood City, CA, USA). The devices were also equipped with software capable of generating a log of the applications used (AppUsage; Benc Software Production, Slavonski Brod, Croatia).

**Figure 2:**
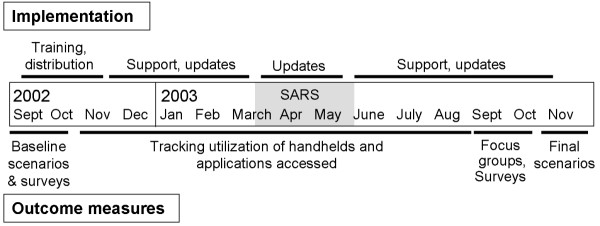
The study time course.

Between September and November 2002 the handheld devices were distributed to participating physicians, at which time they each received a 1-hour training session on the use of the handheld device and the internet link (Fig. 2). After training, the participants were able to utilize the devices in clinical practice for 12 months. We provided 24-hour support by telephone and e-mail, with a website for independent review.

### Outcome measures

#### Feasibility

Feasibility of the system was assessed by tracking physicians' use of the handheld device and tracking their access of the individual handheld applications during the study period. Physicians who updated their handheld computers at least once a month for 6 months were identified as 'regular users'. A qualitative assessment of the system was achieved through surveys and focus group methodology. Participants completed surveys at baseline to identify their prior familiarity with handheld devices, and at the end of the study period to evaluate subjectively the handheld reference system and the individual handheld applications. Survey data were scored on a 7-point scale, in which 'poor' scored 1 and 'excellent' scored 7. An independent company (The NRC+Picker Group, Markham, Canada) conducted the focus group evaluations at the end of the intervention period, to determine the perceived utility of the information system. Each hospital physician group participated in one focus group meeting.

#### Information access

Information sources that physicians accessed to make clinical decisions were evaluated during simulated patient care scenarios, completed in the physicians' own ICU utilizing a computerized patient simulator (SimMan; Laerdal Medical Corporation, Wappingers Falls, NY, USA). Each physician completed one scenario before the handheld device was introduced (baseline scenario) and one at the end of the intervention period (final scenario), when the handheld device could be used (Fig. 2). A small pool of five scenarios with equivalent complexity was developed, such that physicians would likely need to access information sources in order to make management decisions. The scenarios involved unusual but important conditions, namely thyroid storm, myasthenia gravis, methanol toxicity, malaria and methemoglobinaemia. They were allocated to study participants in such a way as to avoid participants from the same site receiving the same scenario at the same time point, and to avoid repetition of scenarios among individual participants. Each scenario concluded with the physician writing admission orders for the simulated patient.

During the scenarios we tracked all medical reference sources utilized by the physicians, who were encouraged to use a 'think aloud' process [15]. An audiovisual recording was made of the scenarios for later analysis, and when the handheld was used real-time screen capture was incorporated into the recording (Additional file 2). This allowed us to document which handheld applications were accessed, the time taken to access information and the time taken to complete the scenario. We developed an objective scoring system for the admission orders generated at each scenario. The admission orders were assigned a score (range 0–100) by a critical care physician (SM) and critical care pharmacist (LB), who were blinded as to whether the physician used the handheld device. The scenario-specific scoring system allocated points for all necessary diagnostic and therapeutic interventions, weighted according to relative importance. Negative points were given for potentially harmful orders.

### Data analysis

Data are presented as median and interquartile range (IQR), and permutation tests were used for comparisons because numbers were small and not normally distributed. The differences between the final and baseline admission order scores and the time to completion of scenarios were calculated for each participant. A two-sample permutation test was used to compare these differences between the group of physicians who chose to use the handheld in the final scenario and those who did not use the device. Admission order scores obtained for each of the five scenarios were compared. Outcomes were considered statistically significant at α < 0.05. The SAS System for Window version 8.2 (SAS Institute, Inc., Cary, NC, USA) was used for all analyses.

Focus groups were recorded, transcribed verbatim and subsequently analyzed. Themes were identified and unique perspectives on key issues noted [16].

## Results

### Feasibility

The handheld information system functioned well during the study period. Tracking of the deployment of handhelds identified 10 regular users (59%), four physicians (23%) who used the system variably and three physicians (18%) who never used their handheld device. The regular users accessed the personal information management applications more commonly (median 32.8 times/month, IQR 28.3–126.8) than the medical software (median 9/month, IQR 3.7–13.7; *P *= 0.028), although significant variation was noted (Table 1).

Baseline survey data identified that, of the 17 critical care physicians participating, 12 (71%) had previous experience with handheld devices (nine had used the Palm operating system, and three had used Windows CE) for a median duration of 1 year (range 1 month to 3.8 years). Seven participants (41%) reported using handhelds for accessing medical information before the study. Of the 16 final survey respondents, seven (44%) felt that the handheld system had had a positive impact on their clinical practice. The handheld medical applications (Critical Care, What's New, Medcalc and PEPID) received similar ratings, with overall evaluation scores ranging from 4.1 to 5.3 on the 7-point scale.

Four focus group meetings, involving a total of 13 participants (76%), identified the benefits and barriers to use of handhelds for information access, and made suggestions for improvement (Table 2). The overall impression of participants was that there is a role for handhelds for mobile information access, but that in situations away from the bedside other electronic media such as desktop computers were preferable.

### Information access

Not all study physicians were able to participate in the simulated clinical scenarios on the pre-assigned day. Fourteen physicians (82.3%) participated in the baseline scenarios and 13 (76.5%) in the final scenarios. Information sources utilized during the baseline scenarios included the internet (50% of participants; e.g. Medline searches and electronic textbooks), textbooks (43%), telephoning colleagues, the ICU pharmacist or Poison Control Centre (71%), and other sources such as pocket guides (21%). In the final scenarios, the handheld device was used as the primary source of information by eight participants (62%; Table 3). Of 14 information searches on the handheld device, 11 searches (79%) were successful and the median time to access information was 19 s (IQR 15–40 s). The information sources of those participants not using the handheld device were similar to those in the baseline surveys (Table 3). Analysis of the time to completion of the clinical scenarios demonstrated no significant difference between those physicians who used the handheld and those who did not (12.92 min, IQR 10.73–16.62 min versus 15.5 min, IQR 12.85–22.72 min, respectively).

**Figure 3:**
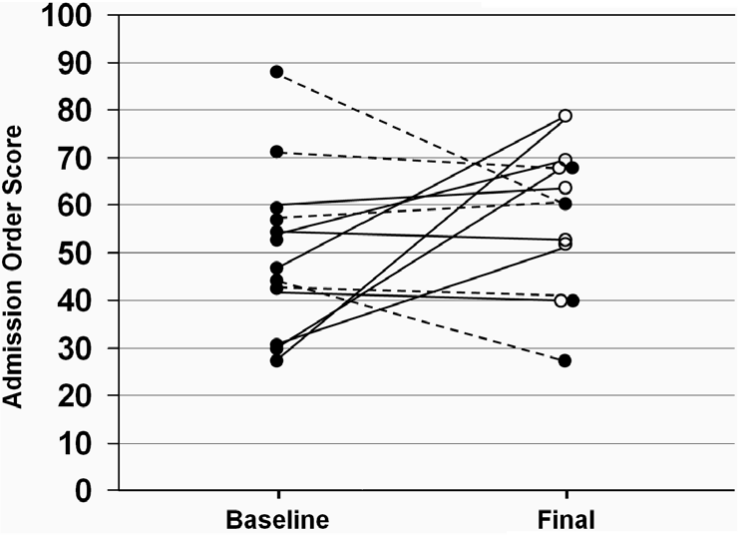
Comparison of scores for admission orders generated during the baseline and final clinical scenarios. Solid lines connect baseline and final scenario scores of participants who used the handheld device in the final scenario, and dotted lines connect scores of participants who did not use the handheld device (solid circles = scenarios where handheld was not used; open circles = scenarios where the handheld device used). A significant improvement was noted in scores in the handheld group as compared with the nonhandheld group (*P *= 0.018).

Physicians who did not use their handheld device in the final clinical scenarios had similar scores to their baseline scenario scores (median 60.0, IQR 40.0–60.0 versus 58.0, IQR 44.5–70.5, respectively). In contrast, an improvement in the final scenario score as compared with the baseline score was noted for those participants who chose to use the handheld device (median 66.0, IQR 52.5–74.5 versus 44.8, IQR 30.5–54.5, respectively; *P *= 0.018; Fig. 3). When scores recorded for each of the five clinical scenarios were compared, no significant difference was noted, reducing the likelihood that scenario assignment influenced outcomes.

## Discussion

This study demonstrates the feasibility of using an electronic knowledge translation system to provide high quality, regularly updated medical reference information from a central academic centre to multiple peripheral users. User acceptance of this technology was not uniform, with just over half of the participants using their handheld devices to access information on a regular basis. Nevertheless, the availability of point-of-care access to information may have improved the quality of clinical decision-making.

Although mobile computing devices have potential beneficial roles to play in clinical medicine, few publications describe formal evaluation of this technology [13]. Because the present study was an early hypothesis-generating evaluation of this technology, multiple quantitative and qualitative outcomes were measured. We generated novel data on the use of handheld devices in a clinical situation, but the study has several limitations. The number of physicians involved was relatively small, with a significant proportion not utilizing the technology. The allocation of clinical scenarios was not randomized, because they were allocated predominantly to avoid using the same scenario at the same site and time point. However, the analysis performed compared participants who used the handheld with those who did not; because it was not known which participant would use the handheld at the time of allocation of scenarios, potential bias was minimized. Furthermore, the scenarios appeared to be equivalent in difficulty because no difference was noted when scores for the individual scenarios were compared. A confounding factor in the study was the outbreak of SARS (severe acute respiratory syndrome) from March to May 2003, which had a significant impact on the study ICUs [17]. Participants were advised to avoid using their handhelds during patient contact because of the potential to transmit infection, and this affected continuity of the study. Had we not encountered this event, utilization might have been higher.

The lack of universal acceptance of this technology is not surprising and may be due to a number of factors, including inadequate training and the lack of familiarity with the technology [18]. Training is essential when introducing handheld computing technology [19,20] and, although all users underwent a training programme, the surveys and focus groups indicated a need for improvement. Familiarity with handhelds is increasing, with 33% of all Canadian physicians and 53% of under 35-year-olds using these devices in 2003, but these levels of utilization remain relatively low when compared with use of the internet, at 88% [21]. Increasing familiarity with the technology will probably increase acceptance of such a system. Other potential barriers to use of the handheld system may be addressed by the rapidly developing technology, including improved screen resolution, ease of data entry and wireless connectivity. Acceptance may be increased through the development of an all-in-one package on the handheld, allowing additional functionality such as decision support, billing, electronic prescribing and communication.

The study demonstrated the potential role of an updateable handheld information system for knowledge translation in critical care. Rapid access to current clinical guidelines may be a valuable component of a comprehensive solution to reducing error and improving efficiency. Information access may be most beneficial in areas without full-time critical care physicians, particularly given the current imbalance between demand and supply with critical care physicians, which is expected to worsen [9,10]. Recent recommendations highlight the importance of leveraging information technology to standardize practice and promote efficiency in critical care [10]. Handheld information access alone is unlikely to change clinical practice, but it should be considered a component of an electronic knowledge translation system. In many situations other media, such as desktop or tablet computers, may be preferable for information access.

Although the study was carried out in a critical care environment, such a system is probably applicable to other specialties in which clinicians are mobile and may not have ready access to a desktop computer (for example, anaesthesia, emergency medicine, home care). This study provides insight into the potential impact of this technology in improving health care outcomes [14]. Nevertheless, further study that builds on our findings is essential to determine how these new technologies can best be incorporated into the patient care setting.

## Conclusion

A handheld computer system is feasible as a means of providing point-of-care access to medical reference material in the ICU. During this study acceptance of this system was variable, and improved training and more advanced technology may be required to overcome some of the barriers we identified. In clinical simulations, use of such a system appeared to improve clinical decision-making.

## Key messages

• 	This study demonstrated that an updateable handheld computer information resource is a feasible means for providing point-of-care access to medical reference information in the ICU.

• 	Acceptance of this system was variable and may be improved by enhanced training and newer technological innovations.

• 	In clinical simulations, this system appeared to improve clinical decision making.

## Competing interests

The author(s) declare that they have no competing intrests.

## Author contributions

Stephen Lapinsky, Randy Wax and Thomas Stewart were responsible for study design. Stephen Lapinsky, Randy Wax, Randy Showalter and Carlos Martinez implemented the handheld system and collected study data. Sangeeta Mehta and Lisa Burry were responsible for data collection and interpretation. Stephen Lapinsky and David Hallet analyzed the data. The manuscript was written by Stephen Lapinsky, Randy Showalter and Thomas Stewart, with all authors participating in revisions and giving approval to the final draft for submission for publication.

## Abbreviations

ICU = intensive care unit; IQR = interquartile range.

## Supplementary Material

Additional File 1 Quicktime movie (video clip) providing a brief overview of the content of the handheld 'Critical Care' handbook, which is used as one of the medical reference sources in the present study. Click here for file

Additional File 2 Quicktime movie (video clip) demonstrating a clinical simulation scenario, using the patient simulator Sim-Man. The physician can be seen accessing the handheld device, and utilization of the various information resources can be tracked. Click here for file

## Figures and Tables

**Table 1:** Prospective tracking of the utilization of handheld applications

Handheld application	Number of accesses/month	
	Median	IQR
Personal information management		
Date book	11.7	1.6–47.7
Address book	8.9	1.5–48.7
To Do List	9.8	4.0–17.7
Note Pad	6.0	2.3–11.3
Memo Pad	0.4	0–4.0
Medical information		
iSilo (Critical Care, Whats New)	3.0	1.5–5.6
Med Calc	0.9	0.4–1.3
PEPID	0.2	0–4.2

Data were collected from 10 participants who used their handheld devices on a regular basis (i.e. updated their handheld device at least monthly for 6 months) IQR, interquartile range.

**Table 2:** Major themes identified during focus group discussions

Theme	Details
Benefits of handheld system	Small size and portability
	Pharmaceutical information
	Literature updates
Preferences for information content	Require more specialty (critical care)-specific content
	Require more practical treatment-based information
	Prefer all content in a single application
Barriers to the use of handhelds	Small text fonts for reading
	Technical problems, predominantly battery discharge
	Inability to access information rapidly:
	Inadequate search engine
	Unfamiliarity with layout of content
	Errors during text entry using handwriting recognition
	Prefer 'all-in-one' solution (e.g. pager, e-mail, physician billing)
Comparison with other information resources	Desktop computer often preferable
	Preferred desktop information resources
	PubMed (Medline literature search)
	Google (internet search engine)
	UpToDate (electronic textbook)

**Table 3:** Evaluation of information sources used during the final clinical scenarios

Resources	Handheld used^a ^(*n *= 8)	Handheld not used^a ^(*n *= 5)
Nonhandheld resources		
UpToDate^b^	1	3
Textbook	0	1
Pharmacy/Poison Control	3	2
Telephone consult	3	3
Mean resources per scenario	0.88	1.8
Handheld resources		
PEPID	11	0
Critical care	2	0
Other	1	0
Mean resources per scenario	1.75	0

^a^The decision to use the handheld device was at the discretion of the individual physician.

^b^UpToDate electronic textbook .
